# Safety of Sabin-Strain Inactivated Poliovirus Vaccine Administered Alone or Concomitantly with Other Vaccines: A Population-Based Post-Marketing Surveillance Study

**DOI:** 10.3390/vaccines14030241

**Published:** 2026-03-06

**Authors:** Lin Chang, Yuxi Liu, Yuan Ren, Jing Li, Xing Fang, Yurong Li

**Affiliations:** 1 Liaoning Provincial Center for Disease Control and Prevention, Shenyang 110172, China; lncdcchanglin@163.com (L.C.); liuyuxideyouxiang@163.com (Y.L.); 2 Sinovac Holding Group Co., Ltd., Beijing 100085, China; renyuan@sinovac.com; 3 Sinovac Biotech Co., Ltd., Beijing 100085, China; lij@sinovac.com

**Keywords:** Sabin-strain inactivated poliovirus vaccine, adverse event following immunization, concomitant vaccination, vaccine safety, surveillance study, real-world evidence

## Abstract

**Background/Objectives**: Sabin-strain inactivated poliovirus vaccine has been increasingly incorporated into routine immunization programs as part of the global strategy to eradicate poliomyelitis. As childhood immunization schedules become more complex, concerns persist regarding the safety of concomitant vaccination. Although randomized controlled trials and regional surveillance studies have demonstrated acceptable safety profiles, additional population-based real-world evidence remains valuable for evaluating the safety of sIPV administered concomitantly with other vaccines under routine programmatic conditions. **Methods**: A retrospective observational study was conducted using vaccination records and adverse events following immunization surveillance data collected in Liaoning Province, China, between 1 January 2022 and 30 June 2025. All reported adverse events following immunization following Sabin-strain inactivated poliovirus vaccine administration were extracted from the Chinese National AEFI Surveillance System. The reporting rates were calculated per 100,000 administered doses. Multivariable Poisson regression models with robust variance estimation were used to estimate adjusted rate ratios and 95% confidence intervals comparing standalone and concomitant sIPV administration, adjusting for sex, age in months, dose number, and city. Interaction analyses between vaccination mode and dose number were additionally performed. **Results**: A total of 205,576 sIPV doses were administered, including 144,724 doses administered alone and 60,852 doses administered concomitantly with other vaccines. Fifty-six adverse events following immunization were reported, corresponding to an overall reporting rate of 27.24 per 100,000 doses. Most reported adverse events following immunization were general reactions (91.07%), and all occurred within seven days after vaccination. The reporting rates for sIPV administered alone and concomitantly were 26.26 and 29.58 per 100,000 doses, respectively, with no significant difference between groups (*p* = 0.7869). After adjustment, concomitant sIPV administration was not associated with an increased risk of adverse events following immunization compared with standalone administration (adjusted rate ratios = 1.13, 95% confidence intervals: 0.59–2.16). No significant interaction between vaccination mode and dose number was identified. **Conclusions**: Sabin-strain inactivated poliovirus vaccine demonstrated a favorable safety profile when administered either alone or concomitantly with other vaccines. These findings support the continued use of flexible and synchronized vaccination strategies involving Sabin-strain inactivated poliovirus vaccine in routine immunization programs.

## 1. Introduction

Poliomyelitis remains a global public health concern despite remarkable progress achieved through the Global Polio Eradication Initiative (GPEI) [1,2]. As the world approaches the final stages of eradication, maintaining high population immunity while minimizing vaccine-associated risks has become increasingly critical [1,2]. In this context, the transition from oral poliovirus vaccine (OPV) to inactivated poliovirus vaccine (IPV) represents a cornerstone strategy to eliminate vaccine-associated paralytic poliomyelitis (VAPP) and reduce the risk of circulating vaccine-derived poliovirus (cVDPV) [1,2,3,4,5].

sIPV is manufactured using attenuated Sabin strains as seed viruses and was developed in the context of the polio endgame to address biosafety and biocontainment concerns related to the production and handling of wild polioviruses required for conventional wild-type inactivated poliovirus vaccine (wIPV) manufacture, as highlighted by World Health Organization Strategic Advisory Group of Experts on Immunization (WHO SAGE) policy guidance [6]. Clinical trials and early post-licensure studies of Sinovac Sabin-strain inactivated poliovirus vaccine (sIPV) have demonstrated that sIPV provides immunogenicity comparable to conventional wIPV, with an acceptable safety profile [7,8,9]. Since its licensure in China in 2021, sIPV has been progressively integrated into routine immunization programs and administered on a large scale.

In routine childhood immunization practice, concomitant administration of multiple vaccines during a single visit is widely implemented to improve coverage, reduce missed opportunities for vaccination, and enhance programmatic efficiency [10]. However, concerns persist among healthcare providers and caregivers regarding the potential accumulation of AEFIs when multiple vaccines are administered simultaneously, which may contribute to vaccine hesitancy and delayed vaccination [11,12]. These concerns are particularly salient during periods of immunization schedule adjustment, including the expansion of IPV use and its alignment with other routine childhood vaccines, especially in the context of gradual transitions toward a full four-dose IPV schedule.

Although clinical trials and early post-licensure studies suggest that concomitant administration of sIPV with other vaccines is generally well tolerated [13,14,15], population-based real-world evidence derived from post-marketing surveillance remains limited, particularly for direct comparisons between standalone and concomitant administration. Passive AEFI surveillance systems play a crucial role in detecting rare or unexpected adverse events under routine conditions, especially for newly introduced vaccines or modified immunization strategies [16,17].

In China, the National Adverse Event Following Immunization (AEFI) Surveillance System provides comprehensive nationwide monitoring of vaccine safety and has generated valuable real-world evidence for multiple vaccines included in the National Immunization Program [4,18]. Although several randomized controlled trials and post-marketing surveillance studies—including recent real-world analyses—have demonstrated acceptable safety of concomitant sIPV administration from different manufacturers, additional population-based data from different regions and programmatic contexts remain valuable to further validate the consistency of these findings [19,20,21].

To address this evidence gap, a population-based analysis of AEFI surveillance data and vaccination records from Liaoning Province, China, between January 2022 and June 2025 was performed. The study hypothesis was that concomitant administration of sIPV with other routine vaccines would not increase the risk of adverse events following immunization compared with standalone administration under routine programmatic conditions. This hypothesis was evaluated by comparing AEFI reporting rates between the two vaccination modes, thereby assessing the real-world safety profile of sIPV and supporting the implementation of synchronized immunization strategies.

## 2. Materials and Methods

### 2.1. Design and Data Sources

This retrospective observational study was conducted using vaccination records and adverse event following immunization (AEFI) surveillance data collected in Liaoning Province, China. Data on sIPV administration and reported AEFIs between 1 January 2022 and 30 June 2025 were extracted from the National Immunization Information System and the Chinese National AEFI Surveillance System, respectively. All data were anonymized prior to analysis. Ethical review and informed consent were waived, as the study involved secondary analysis of routine public health surveillance data in accordance with national regulations.

### 2.2. Vaccine and Immunization Schedule

The Sabin-strain-based inactivated poliovirus vaccine (Vero-cell-derived), manufactured by Sinovac, was administered as a 0.5 mL intramuscular injection. All vaccine batches were released only after passing quality testing conducted by the National Institutes for Food and Drug Control of China.

According to the Chinese National Immunization Program, the poliovirus vaccination schedule consists of two doses of inactivated poliovirus vaccine administered at 2 and 3 months of age, followed by two doses of oral poliovirus vaccine at 4 months and 4 years of age. In routine practice, sIPV may be administered either alone or concomitantly with other vaccines, including both National Immunization Program vaccines and selected non-program vaccines.

During the study period, AEFIs were monitored following all administered doses of sIPV, including the first through fourth doses. Although the routine schedule specifies two IPV doses, sIPV may also be administered as a substitute for oral poliovirus vaccine under certain circumstances, such as catch-up vaccination or self-funded immunization. In this study, “dose number” refers specifically to the sequence of sIPV administration (first, second, third, or fourth dose), regardless of whether the dose was part of the routine schedule or administered as a substitute. All reported AEFIs following any sIPV dose during the study period were included in the analysis.

### 2.3. AEFI Surveillance and Case Classification

AEFI surveillance encompassed all adverse events reported following sIPV administration, including general reactions, abnormal reactions, coincidental events, psychogenic reactions, vaccine-quality-related events, and immunization errors. All reported AEFIs were systematically investigated and classified by local and provincial Centers for Disease Control and Prevention or expert committees, in accordance with national AEFI surveillance guidelines, which are consistent with the World Health Organization (WHO) AEFI surveillance framework [16,22].

According to the underlying cause, AEFIs were categorized into five types: adverse reactions (including general and abnormal reactions), vaccine-quality-related events, immunization errors, coincidental events, and psychogenic reactions [22].

### 2.4. Exposure Definition

sIPV administration was categorized into two exposure groups: (1) sIPV administered alone, and (2) sIPV administered concomitantly with one or more other vaccines during the same vaccination visit. If multiple vaccines were administered concomitantly during a single visit, each vaccine combination was counted once for descriptive analysis.

### 2.5. Statistical Analysis

AEFI reporting rates were calculated as the number of reported events per 100,000 administered doses. Categorical variables were compared using the chi-square test or Fisher’s exact test, as appropriate.

Given the low incidence of reported AEFIs, Poisson regression models were applied to estimate adjusted rate ratios (aRRs) and 95% confidence intervals (CIs). Covariates included sex, age in months, vaccine dose number, and city of residence. The number of administered doses was included as an offset term to account for exposure differences. Age was included in the regression models as categorical age groups consistent with routine immunization schedules. Robust standard errors were used to address mild overdispersion.

A multiplicative interaction term between vaccination mode and dose number was introduced into the multivariable Poisson regression model, and statistical significance was evaluated using a robust Wald test.

All analyses were performed using R software (R.4.5.2) and a two-sided *p* value < 0.05 was considered statistically significant.

## 3. Results

### 3.1. Vaccination and Recipient Characteristics

During the study period, a total of 205,576 doses of sIPV were administered in Liaoning Province. Among these, 144,724 doses (70.4%) were administered alone, while 60,852 doses (29.6%) were administered concomitantly with other vaccines.

The demographic characteristics of vaccine recipients are summarized in Table 1. The distribution of sex was comparable between the two groups. Most doses were administered to children aged 2–107 months, with age distributions differing significantly between the standalone and concomitant administration groups (*p* < 0.001), reflecting routine immunization scheduling practices. Significant differences were also observed across cities and dose numbers (both *p* < 0.001) (Table 1).

### 3.2. Vaccines Administered Concomitantly with sIPV

Among doses administered concomitantly, acellular diphtheria–tetanus–pertussis vaccine (DTaP) was the most frequently co-administered vaccine, accounting for 66.03% of concomitant vaccinations. Other commonly co-administered vaccines included DTaP–Haemophilus influenzae type b (Hib) combination vaccine, diphtheria–tetanus vaccine, meningococcal A + C vaccine, hepatitis B vaccine, and pentavalent rotavirus vaccine. Each of the remaining vaccines accounted for less than 1% of concomitant administrations (Table 2 and Table S1).

Concomitant vaccines were summarized overall rather than stratified by sIPV dose number, as the primary objective of the present study was to evaluate safety differences between standalone and concomitant sIPV administration. In addition, the number of third and fourth sIPV doses administered during the study period was relatively small; therefore, further stratification by dose would substantially increase table complexity without materially influencing the interpretation of safety outcomes.

### 3.3. Overall AEFI Occurrence

A total of 56 AEFIs were reported following sIPV administration during the study period, including 38 cases after standalone administration and 18 cases after concomitant administration. The overall AEFI reporting rate was 27.24 per 100,000 doses.

Most reported AEFIs were classified as general reactions (91.07%), followed by abnormal reactions (5.36%) and coincidental events (0.97%). All AEFIs occurred within seven days after vaccination. The AEFI reporting rates were 26.26 per 100,000 doses for standalone administration and 29.58 per 100,000 doses for concomitant administration, with no statistically significant difference between groups (*p* = 0.7869) (Table 3).

Based on clinical diagnosis, the majority of reported AEFIs were general reactions, primarily including fever, injection-site redness, swelling, and induration. The overall reporting rate of general reactions was 25.24 per 100,000 doses, with similar rates observed for standalone and concomitant administration (24.18 vs. 26.29 per 100,000 doses; *p* = 0.9015).

Abnormal reactions were rare, with an overall reporting rate of 1.46 per 100,000 doses. These reactions were mainly allergic in nature, including allergic rash and allergic purpura. Coincidental events were infrequent and primarily consisted of respiratory infections.

Two serious AEFIs were identified, including one case of allergic purpura in the concomitant administration group and one case of respiratory infection in the standalone sIPV group, the latter being classified as coincidental following causality assessment. Both cases recovered fully without sequelae (Table 3 and Table S2).

The majority of reported AEFIs occurred within three days after vaccination. General reactions such as fever, redness, and induration were most commonly reported within 30 min to three days following sIPV administration. Few events were reported between four and seven days after vaccination, and no AEFIs were reported beyond seven days.

Both abnormal reactions and coincidental events also occurred within the first week after vaccination, with no delayed-onset safety signals observed (Table 3 and Table S3).

### 3.4. AEFI by Sex, Age Group, and City

AEFI reporting rates were similar between male and female recipients. Differences in AEFI reporting rates across most age groups were not statistically significant, except for the 48–59-month age group, in which a significant difference was observed (*p* = 0.005). No significant differences in AEFI reporting rates were identified across cities (Table 4).

Age (months) at each dose by vaccination mode is summarized in Table S4.

### 3.5. AEFI by Dose Number

AEFI reporting rates varied by dose number. The reporting rates were 36.36 per 100,000 doses for the first dose, 20.02 per 100,000 doses for the second dose, and 146.15 per 100,000 doses for the fourth dose. No AEFIs were reported following the third dose. Differences between standalone and concomitant administration were not statistically significant for any dose number (Table 5).

### 3.6. Poisson Regression Analysis

Given the low incidence of reported AEFIs, Poisson regression models were applied. After adjustment for sex, age in months, dose number, and city, concomitant administration of sIPV was not associated with an increased risk of AEFI compared with standalone administration (aRR = 1.13, 95% CI: 0.59–2.16; *p* = 0.71).

Dose number was significantly associated with AEFI reporting, with a lower risk observed for the second dose and a higher risk observed for the fourth dose compared with the first dose. Mild overdispersion was detected and accounted for using robust standard errors, suggesting that the Poisson model provided a reasonable approximation of the data. (Table 6 and Table S5).

### 3.7. Interaction and Dose-Stratified Analyses

To evaluate whether the association between vaccination mode and AEFI reporting differed by dose number, an interaction term between vaccination mode and dose number was included in the multivariable Poisson regression model.

In the interaction model, standalone administration was not significantly associated with AEFI risk at the reference dose (dose 1) (aRR = 1.13, 95% CI: 0.41–3.14, *p* = 0.81). None of the interaction terms were statistically significant (dose 2: *p* = 0.48; dose 4: *p* = 0.22). No interaction estimate was interpretable for dose 3 due to the absence of AEFI events. The overall robust Wald test for interaction was not significant (χ^2^ = 3.17, df = 3, *p* = 0.366).

Dose-stratified analyses were also conducted. For doses 1 and 2, no statistically significant differences in AEFI reporting rates were observed between standalone and concomitant administration after adjustment for age group, sex, and city. No AEFIs were reported following dose 3 in either group during the study period; therefore, a dose-specific adjusted rate ratio could not be estimated. For dose 4, estimates were based on a small number of events and wide confidence intervals (Table S6).

## 4. Discussion

### 4.1. Principal Findings

This population-based surveillance study—the first of its kind conducted in Liaoning Province—demonstrated that the overall reporting rate of AEFIs after either standalone or concomitant administration of sIPV was low. Compared with previous safety surveillance analyses focusing on single-vaccine administration [23,24,25,26,27,28,29], the present study provides a more comprehensive evaluation by fully leveraging available data to assess the real-world safety of concomitant vaccination. Furthermore, in contrast to earlier studies that relied primarily on descriptive analyses of AEFI data [23,24,25,26,27,28,29,30], we employed more rigorous statistical approaches by adjusting for potential confounders and examining interaction effects. These methodological enhancements strengthen the robustness of our findings and provide additional real-world evidence supporting the safety profile of Sinovac sIPV in Anhui province, China [21].

Among more than 200,000 administered doses, only 56 AEFIs were reported, yielding an overall reporting rate of 27.24 per 100,000 doses, which was lower than the national IPV AEFI reporting rate reported in China in 2023 (44.31 per 100,000 doses) [31] and the national surveillance data reported in China from 2021 to 2022 (32.52 per 100,000 doses [29].

Consistent with established temporal patterns of vaccine-related adverse events, all reported AEFIs occurred within seven days after vaccination, highlighting the first post-vaccination week as a critical surveillance window [21,23]. The majority of events were mild, self-limited general reactions, further supporting the favorable safety profile of sIPV under routine immunization conditions.

Importantly, no statistically significant difference in AEFI risk was observed between standalone and concomitant administration, even after adjustment for age, sex, dose number, and geographic region. These results are consistent with the study hypothesis that concomitant administration of sIPV does not increase the risk of adverse events following immunization compared with standalone administration under routine programmatic conditions. Together, these findings provide reassuring real-world evidence supporting the safety of concomitant sIPV administration in routine childhood immunization programs.

### 4.2. Comparison with Previous Studies

The AEFI reporting rate observed in this study was lower than the sIPV-specific AEFI surveillance data from Jiangsu Province (53.02 per 100,000 doses) [30] and the corresponding findings from Anhui Province [21]. The comparatively lower reporting rate in our study may be attributable to the relatively smaller vaccine utilization in the study setting or to differences in surveillance sensitivity across regions.

The findings of this study are consistent with previous post-marketing surveillance and observational studies conducted in multiple provinces across China, including Jiangxi, Jilin, Shanxi, and Hebei, which have reported low AEFI rates and predominantly mild clinical manifestations following IPV or sIPV vaccination [23,24,25,26,27]. Similar to these reports, the majority of AEFIs identified in the present study were classified as general reactions, such as fever, local redness, swelling, and induration, with serious or abnormal reactions being rare, and all reported serious vaccine-related cases recovered fully without long-term sequelae.

The overall AEFI reporting rate observed in Liaoning Province was comparable to that reported in Jiangxi Province [23] and slightly lower than that reported in Jilin Province [24], further reinforcing the consistency of sIPV safety profiles across different geographic regions. No long-term sequelae were observed in any reported serious cases, which aligns with previous findings indicating that serious AEFIs associated with IPV are rare and generally reversible [25,26,27].

### 4.3. Concomitant Vaccination and Safety

Concomitant vaccination has become an essential strategy for maintaining high immunization coverage, particularly in settings with increasingly complex childhood immunization schedules. In this study, no significant differences were observed between standalone and concomitant administration groups in terms of overall AEFI incidence, general reaction rates, or abnormal reaction rates, findings that are consistent with previous research conducted in Jilin Province [24] and other domestic studies [28].

The Poisson regression analysis further confirmed the absence of an increased AEFI risk associated with concomitant vaccination (aRR = 1.13, *p* = 0.7113), indicating that simultaneous administration of sIPV with other routine childhood vaccines does not elevate safety risks. These findings are consistent with previous evidence reported from Anhui Province [21]. Given the extremely low absolute incidence of AEFIs observed in both groups, these results provide strong empirical support for the continued implementation of concomitant vaccination strategies.

The interaction analysis provided no evidence of effect modification by dose number, indicating that the association between vaccination mode and AEFI reporting was stable across sIPV doses. The non-significant overall interaction test supports the robustness of the primary findings. Estimates for later doses should be interpreted cautiously given the small number of observed events.

In China, recent adjustments to the diphtheria–tetanus–pertussis (DTaP) immunization schedule have resulted in greater temporal overlap with IPV doses. The present findings offer timely real-world safety evidence supporting such synchronized vaccination strategies, which aim to reduce clinic visits, improve caregiver compliance, and enhance immunization service efficiency.

### 4.4. Dose-Specific and Age-Specific Findings

Dose-specific analysis revealed that the risk of AEFIs following the second dose was significantly lower than that following the first dose, consistent with the commonly observed phenomenon of higher reactogenicity after initial vaccine exposure [32]. Previous prospective surveillance studies have also demonstrated a decreasing trend in adverse event incidence across IPV doses, with the first dose exhibiting the highest rate and subsequent doses showing lower rates, a pattern attributed to increased tolerability following initial exposure [33]. In the present passive surveillance study, no AEFIs were reported after the third dose. Given the extremely low overall reporting rate and the limited number of later-dose recipients, this likely reflects sparse data and random variation rather than a true absence of risk. Although a higher reporting rate was observed for the fourth dose, this estimate was based on very few events and wide CIs, and should therefore be interpreted cautiously.

Age-stratified analysis showed a statistically significant difference in AEFI reporting rates among children aged 48–59 months, consistent with findings from some previous surveillance studies [25,26]. This observation may be influenced by the immunization schedule currently adopted in Liaoning Province, in which two doses of IPV are administered followed by two doses of OPV, leading to a relatively small number of children receiving the third and fourth IPV doses. This markedly elevated aRR should not be interpreted as evidence of increased biological risk but rather reflects sparse data and statistical instability inherent to rare-event analyses.

Given the substantial age heterogeneity across dose numbers—particularly for the second dose—potential age-related confounding was carefully evaluated. However, adjustment for age in multivariable models and dose-stratified analyses did not materially alter the effect estimates, indicating that age was not a major confounding factor in the association between vaccination mode and AEFI reporting.

### 4.5. Strengths and Limitations

This study has several notable strengths, including its large population base, extended observation period, and use of real-world surveillance data reflecting routine immunization practices. The direct comparison between standalone and concomitant administration enhances the practical relevance of the findings for immunization policy and clinical decision-making.

Nevertheless, several limitations should be acknowledged. As a passive surveillance system, AEFI monitoring relies heavily on voluntary reporting, which may lead to underreporting, particularly for mild or transient reactions [34]. Reporting sensitivity may also be influenced by public awareness, media coverage, and evolving perceptions of vaccine safety, potentially introducing reporting bias [35,36]. In addition, causal inference is inherently limited in observational studies based on passive surveillance data. Additionally, the concomitant vaccination group comprised heterogeneous vaccine combinations, precluding vaccine-specific risk estimation.

Despite these limitations, passive surveillance remains an indispensable tool for post-marketing vaccine safety evaluation, particularly for identifying rare or unexpected adverse events.

### 4.6. Implications

Despite the inherent limitations of passive surveillance, the findings of this study provide robust real-world evidence supporting the safety of sIPV administered either alone or concomitantly with other routine vaccines. The absence of an increased AEFI risk associated with concomitant administration may help alleviate concerns among healthcare providers and caregivers and supports the continued use of flexible, synchronized immunization schedules. When considered alongside previously Sinovac’s published immunogenicity non-inferiority trial [13], these real-world safety data further strengthen the overall evidence base for concomitant Sinovac’s sIPV use under routine immunization program conditions.

Future studies integrating active surveillance systems and retrospective cohort analyses are warranted to further validate these findings and to provide a more comprehensive assessment of sIPV safety across different populations and immunization settings.

## 5. Conclusions

In conclusion, this population-based surveillance study demonstrated that the Sabin-strain inactivated poliovirus vaccine exhibited a favorable safety profile when administered either alone or concomitantly with other vaccines. The overall reporting rate of adverse events following immunization was low, and most reported events were mild and occurred shortly after vaccination. Concomitant administration was not associated with an increased risk of adverse events following immunization compared with standalone administration. These findings provide real-world evidence supporting the continued use of flexible and synchronized sIPV vaccination strategies within routine immunization programs.

## Figures and Tables

**Table 1:** Baseline demographic and vaccination characteristics of individuals receiving sIPV administered alone or concomitantly with other vaccines in Liaoning Province, China (2022–2025).

Characteristic	sIPV Alone (Nn = 144,724)	Concomitant sIPV (N = 60,852)	Total (N = 205,576)	*p*-Value
Sex, n (%)				0.001
Male	74,481 (51.46)	31,826 (52.30)	106,307 (51.70)	
Female	70,243 (48.54)	29,026 (47.70)	99,269 (48.30)	
Age group, months, n (%)				<0.001
2	29,994 (20.72)	5824 (9.57)	35,818 (17.42)	
3	12,725 (8.79)	22,193 (36.47)	34,918 (16.99)	
4–17	13,632 (9.42)	19,692 (32.36)	33,324 (16.21)	
18–47	3031 (2.09)	2753 (4.52)	5784 (2.81)	
48–59	11,676 (8.07)	731 (1.20)	12,407 (6.04)	
60–107	73,666 (50.90)	9659 (15.87)	83,325 (40.53)	
City, n (%)				<0.0001
Shenyang	24,025 (16.60)	12,736 (20.93)	36,761 (17.88)	
Anshan	15,636 (10.80)	7957 (13.08)	23,593 (11.48)	
Fushun	4644 (3.21)	1995 (3.28)	6639 (3.23)	
Benxi	3469 (2.40)	1804 (2.96)	5273 (2.56)	
Dandong	4556 (3.15)	2690 (4.42)	7246 (3.52)	
Jinzhou	7162 (4.95)	2272 (3.73)	9434 (4.59)	
Yingkou	8816 (6.09)	5536 (9.10)	14,352 (6.98)	
Fuxin	4316 (2.98)	1473 (2.42)	5789 (2.82)	
Liaoyang	8163 (5.64)	3668 (6.03)	11,831 (5.76)	
Panjin	4863 (3.36)	3890 (6.39)	8753 (4.26)	
Tieling	11,511 (7.95)	3750 (6.16)	15,261 (7.42)	
Chaoyang	31,809 (21.98)	6902 (11.34)	38,711 (18.83)	
Huludao	15,754 (10.89)	6179 (10.15)	21,933 (10.67)	
Dose number, n (%)				<0.001
Dose 1	40,674 (28.10)	14,329 (23.55)	55,003 (26.76)	
Dose 2	94,542 (65.33)	40,327 (66.27)	134,869 (65.61)	
Dose 3	5507 (3.81)	4039 (6.64)	9546 (4.64)	
Dose 4	4001 (2.76)	2157 (3.54)	6158 (3.00)	

*p*-values represent comparisons between standalone and concomitant administration groups for each characteristic and were calculated using the chi-square test (or Fisher’s exact test, as appropriate). “Dose number” refers specifically to the sequence of sIPV administration (first through fourth dose), regardless of whether the dose was part of the routine schedule or administered as a substitute.

**Table 2:** Distribution of vaccines administered concomitantly with sIPV.

Concomitant Vaccine	Number (N)	Proportion (%)
DTaP	40,283	66.03
DTaP–Hib combination	4854	7.96
DT	4755	7.79
Meningococcal A + C	3600	5.90
Hepatitis B	3007	4.93
Pentavalent rotavirus	2050	3.36
Other vaccines *	2303	<5

* Includes vaccines each accounting for <1% of concomitant administrations, such as varicella, measles–mumps–rubella, Japanese encephalitis, influenza, pneumococcal, EV71, and others. If more than one vaccine was administered during the same visit, each combination was counted once. Detailed distribution shown in Table S1.

**Table 3:** Overall reporting rates of AEFIs after sIPV administered alone or concomitantly with other vaccines.

Outcome	sIPV Alone (N = 144,724)	Concomitant sIPV (N = 60,852)	Total (N = 205,576)	*p*-Value
Any AEFI	38 (26.26)	18 (29.58)	56 (27.24)	0.7869
Serious AEFI *	1 (0.69)	1 (1.64)	2 (0.97)	0.6336
Time to onset				
≤30 min	3 (2.07)	0 (0.00)	3 (1.46)	0.6236
0–3 days	34 (23.49)	18 (29.58)	52 (25.29)	0.5220
≤7 days	38 (26.26)	18 (29.58)	56 (27.24)	0.7869
AEFI category				
General reactions	35 (24.18)	16 (26.29)	51 (25.24)	0.9015
General reaction symptoms	32 (22.11)	16 (26.29)	48 (23.35)	0.6829
Abnormal reactions	1 (0.69)	2 (3.29)	3 (1.46)	0.4389
Coincidental events	2 (1.38)	0 (0.00)	2 (0.97)	0.8867

Values are presented as number (reporting rate per 100,000 doses). *p*-values represent comparisons between standalone and concomitant administration groups for each outcome and were calculated using the chi-square test or Fisher’s exact test, as appropriate. * Details of the clinical diagnoses of these serious AEFIs are provided in Section 3.7 and Supplementary Table S3.

**Table 4:** Reporting rates of AEFIs by sex and age group after sIPV administration.

Variable	sIPV Alone	Concomitant sIPV	Total	*p*-Value
Sex				
Male	20 (26.85)	9 (28.28)	29 (27.28)	0.897
Female	18 (25.63)	9 (31.01)	27 (27.20)	0.641
Age group (months)				
2	7 (23.34)	1 (17.17)	8 (22.34)	0.774
3	7 (55.01)	6 (27.04)	13 (37.23)	0.202
4–17	2 (14.67)	6 (30.47)	8 (24.01)	0.371
18–47	1 (32.99)	3 (108.97)	4 (69.16)	0.301
48–59	1 (8.56)	2 (273.60)	3 (24.18)	0.005
60–107	20 (27.15)	0 (0.00)	20 (24.00)	1.000
City				
Shenyang	11 (45.79)	3 (23.56)	14 (38.08)	0.308
Anshan	6 (38.37)	3 (37.70)	9 (38.15)	0.980
Fushun	4 (86.13)	2 (100.25)	6 (90.38)	0.861
Benxi	4 (115.31)	0 (0.00)	4 (75.86)	1.000
Dandong	0 (0.00)	5 (185.87)	5 (69.00)	1.000
Jinzhou	1 (13.96)	0 (0.00)	1 (10.60)	1.000
Yingkou	0 (0.00)	1 (18.06)	1 (6.97)	1.000
Fuxin	0 (0.00)	0 (0.00)	0 (0.00)	–
Liaoyang	0 (0.00)	2 (54.53)	2 (16.90)	1.000
Panjin	0 (0.00)	2 (51.41)	2 (22.85)	1.000
Tieling	1 (8.69)	0 (0.00)	1 (6.55)	1.000
Chaoyang	5 (15.72)	0 (0.00)	5 (12.92)	1.000
Huludao	6 (38.09)	0 (0.00)	6 (27.36)	1.000

Values are presented as number (reporting rate per 100,000 doses). *p*-values represent comparisons between standalone and concomitant administration groups within each stratum and were calculated using the chi-square test or Fisher’s exact test, as appropriate.

**Table 5:** Reporting rates of AEFIs by dose number after sIPV administered alone or concomitantly with other vaccines.

Dose Number	sIPV Alone (N = 144,724)	Concomitant sIPV (N = 60,852)	Total (N = 205,576)	*p*-Value
Dose 1	13 (31.96)	7 (48.85)	20 (36.36)	0.365
Dose 2	20 (21.15)	7 (17.36)	27 (20.02)	0.652
Dose 3	0 (0.00)	0 (0.00)	0 (0.00)	1.000
Dose 4	5 (124.97)	4 (185.44)	9 (146.15)	0.556

Values are presented as number (reporting rate per 100,000 doses). *p*-values represent comparisons between standalone and concomitant administration groups for each dose number and were calculated using the chi-square test or Fisher’s exact test, as appropriate.

**Table 6:** aRRs and 95% CIs for AEFIs after sIPV administration, estimated using Poisson regression.

Variable	aRR	95% CI	*p*-Value
Vaccination mode			
Standalone vs. concomitant	1.13	0.59–2.16	0.711
Age group (months)			
3 vs. 2	3.62	1.22–10.78	0.021
4–17 vs. 2	3.05	0.99–9.40	0.053
18–47 vs. 2	0.23	0.02–2.28	0.211
48–59 vs. 2	0.59	0.09–3.97	0.586
60–107 vs. 2	3.00	0.99–9.05	0.052
Sex			
Male vs. female	0.99	0.60–1.64	0.971
Dose number			
Dose 2 vs. dose 1	0.35	0.17–0.73	0.005
Dose 4 vs. dose 1	17.07	3.09–94.24	0.001
City *			
Chaoyang vs. Anshan	0.35	0.13–0.95	0.040
Fushun vs. Anshan	3.02	1.15–7.90	0.024

Rate ratios were adjusted for sex, age in months, dose number, and city, with the number of administered doses included as an offset. *p*-values correspond to robust Wald tests for each regression coefficient in the multivariable Poisson regression model (sandwich variance estimator). * Only cities with statistically significant associations are shown; full results are provided in Supplementary Table S5.

## Data Availability

The data presented in this study are available from the corresponding author upon reasonable request, subject to data protection regulations.

## Supplementary Materials

The following supporting information can be downloaded at: https://www.mdpi.com/article/10.3390/vaccines14030241/s1. The supplementary materials provide additional detailed data supporting the main analyses of this study. Table S1: Detailed Distribution of Vaccines Administered Concomitantly with sIPV. Table S2: aRRs and 95% CIs for AEFIs after sIPV administration, estimated using Poisson regression models. Table S3: Detailed Symptom Distribution of Reported AEFIs after sIPV Administration. Table S4: Clinical characteristics and time to onset of reported AEFIs after sIPV administered alone or concomitantly with other vaccines.

## Abbreviations

The following abbreviations are used in this manuscript:

AEFI	adverse event following immunization
CI	confidence interval
cVDPV	circulating vaccine-derived poliovirus
DT	diphtheria–tetanus vaccine
DTaP	acellular diphtheria–tetanus–pertussis vaccine
GPEI	Global Polio Eradication Initiative
Hib	Haemophilus influenzae type b
IPV	inactivated poliovirus vaccine
OPV	oral poliovirus vaccine
aRR	adjusted rate ratio
sIPV	Sabin-strain inactivated poliovirus vaccine
VAPP	vaccine-associated paralytic poliomyelitis
WHO	World Health Organization
WHO SAGE	World Health Organization Strategic Advisory Group of Experts on Immunization
wIPV	wild-type inactivated poliovirus vaccine

## References

[1] World Health Organization Poliomyelitis. https://www.who.int/news-room/fact-sheets/detail/poliomyelitis.

[2] World Health Organization (2022). Polio Vaccines: WHO Position Paper—June 2022. Wkly. Epidemiol. Rec..

[3] World Health Organization (2021). Polio Eradication Strategy 2022–2026: Delivering on a Promise.

[4] Platt L.R., Estívariz C.F., Sutter R.W. (2014). Vaccine-associated paralytic poliomyelitis: A review of the epidemiology and estimation of the global burden. J. Infect. Dis..

[5] Lai Y.A., Chen X., Kunasekaran M., Rahman B., MacIntyre C.R. (2022). Global epidemiology of vaccine-derived poliovirus, 2016–2021: A descriptive analysis and retrospective case–control study. eClinicalMedicine.

[6] World Health Organization, Strategic Advisory Group of Experts (SAGE) (2022). Evidence-to-Decision: Interchangeability of IPV Vaccines (Including Sabin-Strain IPV).

[7] Hu Y., Wang J., Zeng G., Chu K., Jiang D., Zhu F., Ying Z., Chen L., Li C., Zhu F. (2019). Immunogenicity and safety of a Sabin strain–based inactivated poliovirus vaccine: A phase 3 clinical trial. J. Infect. Dis..

[8] Chu K., Han W., Jiang D., Jiang Z., Zhu T., Xu W., Hu Y., Zeng G. (2021). Cross-neutralization capacity of immune serum from different dosages of Sabin inactivated poliovirus vaccine immunization against multiple polioviruses. Expert Rev. Vaccines.

[9] Kai C., Yurong L., Sheng L., Yongmei S., Jianfeng W., Xinge L., Peng J., Hongxing P. (2025). Sustained immune persistence five years post-completion of four-dose sIPV vaccination. Vaccines.

[10] World Health Organization (2020). Immunization Agenda 2030: A Global Strategy to Leave No One Behind.

[11] Karafillakis E., Larson H.J., ADVANCE Consortium (2017). The benefit of the doubt or doubts over benefits? A systematic literature review of perceived risks of vaccines in European populations. Vaccine.

[12] Larson H.J., Jarrett C., Eckersberger E., Smith D.M.D., Paterson P. (2014). Understanding vaccine hesitancy around vaccines and vaccination from a global perspective: A systematic review of published literature, 2007–2012. Vaccine.

[13] Hu J., Han W., Chu K., Zhang H., Tuo L., Duan X., Li J., Yuan F., Luan C., Pan H. (2025). Immunogenicity and safety of a Sabin strain inactivated poliovirus vaccine booster dose administered separately or concomitantly with inactivated hepatitis A vaccine or measles–mumps–rubella vaccine: A phase 4 randomized controlled trial. Vaccines.

[14] Guo S., Li Z., Zheng M., Wu F., Sun J., Tuo L., Li S., Li X., Wei L., Xia Z. (2024). Safety and 6-month immune persistence of Sabin strain inactivated poliovirus vaccine administered simultaneously with other vaccines. Vaccine.

[15] Zheng M., Li L., Guo S., Mao Q., Kan X., Wei L., Liu J., Zhao Y. (2024). Post-marketing safety surveillance of Sabin strain inactivated poliovirus vaccine administered concomitantly with other vaccines in infants and young children in China. Chin. J. Vaccines Immun..

[16] World Health Organization (2016). Global Manual on Surveillance of Adverse Events Following Immunization.

[17] Moro P.L., Haber P., McNeil M.M. (2019). Challenges in evaluating post-licensure vaccine safety: Observations from the Centers for Disease Control and Prevention. Expert Rev. Vaccines.

[18] Liu D., Wu W., Li K., Xu D., Ye J., Li L., Wang H. (2015). Surveillance of adverse events following immunization in China: Past, present, and future. Vaccine.

[19] Xu Y., Chen H., Wang B., Zhu X., Luo L., Wang S., Xiao Y., Wang H., Ma R., Liu S. (2023). Immunogenicity and safety of concomitant administration of the Sabin-strain-based inactivated poliovirus vaccine, the diphtheria–tetanus–acellular pertussis vaccine, and measles–mumps–rubella vaccine to healthy infants aged 18 months in China. Int. J. Infect. Dis..

[20] Liu X., Han S., Wang H., Sun L., Zhang C., Chen X., Wang R., Chang S., Shi X., Chen H. (2025). Immunogenicity and safety of co-administration of Sabin-strain-based inactivated poliovirus vaccine, diphtheria–tetanus–acellular pertussis vaccine, and live attenuated hepatitis A vaccine in 18-month-old children: A multicenter randomized controlled non-inferiority trial in China. Vaccine.

[21] Wang B., Meng F., Xue W., Su Y., Jiang T., Dong Y., Ren M., Tang J. (2026). Safety of Sabin Inactivated Poliovirus Vaccine Administered Standalone or Concomitantly with Other Childhood Vaccines: A Real-World Study in China. Vaccines.

[22] National Health Commission of the People’s Republic of China, National Medical Products Administration (2022). Notice on Revising Selected Contents of the National Surveillance Program for Suspected Adverse Events Following Immunization.

[23] Zheng M., Wu F., Zhao H., Xu F., Guo S., He W., Cheng H. (2020). Surveillance for adverse events following immunization with inactivated poliovirus vaccine in Jiangxi Province, China, 2016–2019. Chin. J. Vaccines Immun..

[24] Lin L., Fu S., Tian X., Cao F., Sun M., Chen T., Wu Y., Li Y. (2022). Adverse events following immunization with inactivated poliovirus vaccine and acellular diphtheria–tetanus–pertussis vaccine administered alone or concomitantly in Jilin Province, China. Chin. J. Vaccines Immun..

[25] Liu X., Ma R., Hu W., Gao Y., Hou X., Wang H., Zhang S. (2021). Surveillance analysis of adverse events following immunization with Sabin strain inactivated poliovirus vaccine in Shaanxi Province, China, 2018–2020. Chin. J. Biol..

[26] Sun L., Wang S., Wang Y., Wang Y., Wang J., Li J., Xu X., Zhang J., Cong Y., Guo Y. (2024). Surveillance for Adverse Events Following Immunization in Hebei Province, China, 2018–2020. Hum. Vaccines Immunother..

[27] Sun L., Xu X., Wang J., Cong Y., Wang Y., Wang Y., Li J., Zhang Z. (2020). Safety surveillance analysis of three poliovirus vaccines in Hebei Province, China. Pract. Prev. Med..

[28] Liu X., Chen H., Zhang Z., Han S., Shi X., Sun L. (2019). Post-marketing safety evaluation on inactivated poliovirus vaccine (Vero cells) made from Sabin strain. Chin. J. Biol..

[29] Li Y., Li K., Li Y., Fan C., Zhang L., Ren M., Cao L., Yu W., Yin Z. (2025). Post-marketing surveillance of adverse events following rotavirus vaccination—China, 2013–2023. China CDC Wkly..

[30] Kang G., Tang F., Wang Z., Hu R., Yu J., Gao J. (2021). Surveillance of adverse events following the introduction of inactivated poliovirus vaccine made from Sabin strains to the Chinese EPI and comparison with wild-strain IPV in Jiangsu, China. Hum. Vaccin. Immunother..

[31] Zhang L., Li K., Li Y., Fan C., Li Y., Ren M., Cao L., Yu W., Yin Z. (2025). Surveillance of adverse events following immunization in China, 2023. Chin. J. Vaccines Immun..

[32] Hervé C., Laupèze B., Del Giudice G., Didierlaurent A.M., Tavares Da Silva F. (2019). The how’s and what’s of vaccine reactogenicity. npj Vaccines.

[33] Wang M., Guang M., Gao Z.-G., Liu F., Chen H.-P. (2020). Safety Evaluation on Different Doses of Inactivated Poliomyelitis Vaccine. Chin. J. New Drugs.

[34] Rosenthal S., Chen R. (1995). The reporting sensitivities of two passive surveillance systems for vaccine adverse events. Am. J. Public Health.

[35] Luo C., Du J., Cuker A., Lautenbach E., Asch D.A., Poland G.A., Tao C., Chen Y. (2022). Comparability of clinical trials and spontaneous reporting data regarding COVID-19 vaccine safety. Sci. Rep..

[36] Eberth J.M., Kline K.N., Moskowitz D.A., Montealegre J.R., Scheurer M.E. (2014). The role of media and the Internet on vaccine adverse event reporting: A case study of human papillomavirus vaccination. J. Adolesc. Health.

