# Supplementary material for: Safety of Sabin-Strain Inactivated Poliovirus Vaccine Administered Alone or Concomitantly with Other Vaccines: A Population-Based Post-Marketing Surveillance Study
Source: Vaccines (Basel). 2026 Mar 6;14(3):241. doi: 10.3390/vaccines14030241 (PMC13030381; doi:10.3390/vaccines14030241)
Supplement: Supplementary file 1 [file vaccines-14-00241-s001.zip › vaccines-4163833-supplementary.pdf]

Table S1. Detailed Distribution of Vaccines Administered Concomitantly with sIPV

Concomitant Vaccine	Number (N)	Proportion (%)
DTaP	40,283	66.03
DTaP–Hib	4,854	7.96
DT	4,755	7.79
Meningococcal A + C	3,600	5.90
Hepatitis B	3,007	4.93
Pentavalent rotavirus	2,050	3.36
Live JE	441	0.72
Varicella	423	0.69
MMR	255	0.42
Rotavirus	237	0.39
Meningococcal ACYW	181	0.30
Meningococcal A	172	0.28
Live Hepatitis A	163	0.27
Inactivated Hepatitis A	130	0.21
Influenza	108	0.18
PCV13 (CRM197)	103	0.17
Hib	65	0.11
Meningococcal A + C (conjugate)	60	<0.1
PPV23	38	<0.1
EV71 (inactivated)	35	<0.1
Cholera	15	<0.1
OPV	9	<0.1
Intranasal influenza	6	<0.1
Mumps	6	<0.1
BCG	5	<0.1
Inactivated JE	4	<0.1
Rabies	3	<0.1

Table S2. Detailed Symptom Distribution of Reported AEFIs after sIPV Administration

Symptom	sIPV Alone	Concomitant sIPV	Total	*p*-Value
**General reaction symptoms**				
Fever	23 (15.89)	11 (18.08)	34 (16.54)	0.8700
37.1–37.5 °C	3 (2.07)	1 (1.64)	4 (1.95)	1.000
37.6–38.5 °C	10 (6.91)	4 (6.57)	14 (6.81)	1.000
≥38.6 °C	10 (6.91)	6 (9.86)	16 (7.78)	0.6757
Injection-site redness	10 (6.91)	2 (3.29)	12 (5.84)	0.5058
≤2.5 cm	6 (4.15)	1 (1.64)	7 (3.41)	0.6357
2.6–5.0 cm	3 (2.07)	1 (1.64)	4 (1.95)	1.000

>5.0 cm	1 (0.69)	0 (0.00)	1 (0.49)	1.000
Induration	6 (4.15)	1 (1.64)	7 (3.41)	0.6357
≤2.5 cm	4 (2.76)	1 (1.64)	7 (3.41)	1.000
2.6–5.0 cm	2 (1.38)	0 (0.00)	4 (1.95)	0.8867
Other general reactions	3 (2.07)	0 (0.00)	3 (1.46)	0.6236
**Abnormal reactions**				
Allergic rash	1 (0.69)	1 (1.64)	2 (0.97)	1.000
Allergic purpura	0 (0.00)	1 (1.64)	1 (0.49)	0.6550
**Coincidental events**				
Respiratory infection	2 (1.38)	0 (0.00)	2 (0.97)	0.8867

Table S3. Clinical characteristics and time to onset of reported AEFIs after sIPV administered alone or concomitantly with other vaccines.

**Outcome**	**sIPV Alone (N = 144,724)**	**Concomitant sIPV (N = 60,852)**	**Total (N = 205,576)**
**Any AEFI**	38 (26.26)	18 (29.58)	56 (27.24)
**General reactions**	35 (24.18)	16 (26.29)	52 (25.29)
General reaction symptoms	32 (22.11)	16 (26.29)	48 (23.35)
Fever	23 (15.89)	11 (18.08)	34 (16.54)
0–30 min	0 (0.00)	1 (1.64)	1 (0.49)
30 min–3 days	22 (15.20)	10 (16.43)	32 (15.57)
4–7 days	1 (0.69)	0 (0.00)	1 (0.49)
Injection-site redness	10 (6.91)	2 (3.29)	12 (5.84)
0–30 min	0 (0.00)	0 (0.00)	0 (0.00)
30 min–3 days	9 (6.22)	2 (3.29)	11 (5.35)
4–7 days	1 (0.69)	0 (0.00)	1 (0.49)
Injection-site induration	6 (4.15)	1 (1.64)	7 (3.41)
0–30 min	0 (0.00)	0 (0.00)	0 (0.00)
30 min–3 days	4 (2.76)	1 (1.64)	5 (2.43)
4–7 days	2 (1.38)	0 (0.00)	2 (0.97)
Other general reactions	3 (2.07)	0 (0.00)	3 (1.46)
0–30 min	1 (0.69)	0 (0.00)	1 (0.49)
30 min–3 days	2 (1.38)	0 (0.00)	2 (0.97)
**Abnormal reactions**	1 (0.69)	2 (3.29)	3 (1.46)
Allergic rash	1 (0.69)	1 (1.64)	2 (0.97)
30 min–3 days	1 (0.69)	1 (1.64)	2 (0.97)
Allergic purpura	0 (0.00)	1 (1.64)	1 (0.49)
30 min–3 days	0 (0.00)	1 (1.64)	1 (0.49)
**Coincidental events**	2 (1.38)	0 (0.00)	2 (0.97)
Respiratory infection	2 (1.38)	0 (0.00)	2 (0.97)

30 min–3 days	1 (0.69)	0 (0.00)	1 (0.49)
4–7 days	1 (0.69)	0 (0.00)	1 (0.49)

Values are presented as number (reporting rate per 100,000 doses). Time to onset was calculated as the interval between vaccination and symptom onset.

Table S4. Age (months) at each dose when the vaccine was administered alone and concomitantly

Dose	Administered Alone			Administered Concomitantly		
Number	Mean Age	Minimum	Maximum	Mean Age	Minimum	Maximum
Dose 1	12.74	2	298	7.86	2	197
Dose 2	64.75	2	151	21.60	2	173
Dose 3	30.29	4	106	8.56	4	131
Dose 4	37.09	18	102	24.10	18	96

Table S5. aRRs and 95% CIs for AEFIs after sIPV administration, estimated using Poisson regression models.

Variable	aRR	95% CI (Lower)	95% CI (Upper)	*p*-Value
**Vaccination mode**				
Standalone vs. concomitant	1.13	0.5916	2.1584	0.7113
**Age group (months)**				
3 vs. 2	3.6223	1.2176	10.7757	0.0207
4–17 vs. 2	3.0463	0.9867	9.4049	0.0528
18–47 vs. 2	0.2334	0.0239	2.2803	0.2109
48–59 vs. 2	0.5878	0.0871	3.9684	0.5855
60–107 vs. 2	2.9961	0.9922	9.0471	0.0516
**Sex**				
Male vs. female	0.9907	0.6001	1.6357	0.9709
**City (reference: Anshan)**				
Benxi	2.3963	0.8539	6.7245	0.0969
Chaoyang	0.3467	0.1262	0.9523	0.0399
Dandong	2.1350	0.5745	7.9338	0.2574
Fushun	3.0182	1.1534	7.8981	0.0244
Fuxin	0.0000	0.0000	0.0000	0.0000
Huludao	0.8174	0.2570	2.6002	0.7327
Jinzhou	0.3105	0.0469	2.0545	0.2251
Liaoyang	0.4101	0.0804	2.0905	0.2834
Panjin	0.6430	0.1450	2.8514	0.5612
Shenyang	1.0988	0.4486	2.6915	0.8367
Tieling	0.2004	0.0342	1.1752	0.0749
Yingkou	0.2257	0.0275	1.8553	0.1661
**Dose number (reference: Dose 1)**				
Dose 2	0.3474	0.1658	0.7278	0.0051

Dose 3	NA	NA	NA	NA
Dose 4	17.0745	3.0936	94.2400	0.0011

Table S6. aRRs for AEFI comparing sIPV administered alone versus concomitantly, stratified by dose number

**Dose Number**	**aRR**	**95% CI**	***p*-Value**
Dose 1	1.19	0.44–3.23	0.7289
Dose 2	1.92	0.85–4.36	0.1176
Dose 3	NA	NA	NA
Dose 4	0.25	0.07–0.85	0.0268

Abbreviations: JE, Japanese encephalitis; MMR, measles–mumps–rubella; PCV13, 13-valent pneumococcal conjugate vaccine.
